# A highly flexible and repeatable genotyping method for aquaculture studies based on target amplicon sequencing using next-generation sequencing technology

**DOI:** 10.1038/s41598-019-43336-x

**Published:** 2019-05-06

**Authors:** Mana Sato, Sho Hosoya, Sota Yoshikawa, Shun Ohki, Yuki Kobayashi, Takuya Itou, Kiyoshi Kikuchi

**Affiliations:** 1 Fisheries Laboratory, University of Tokyo, Hamamatsu, 431-0214 Japan; 2 Seed production Technology Development Center, Nagasaki Prefectural Institute of Fisheries, Nagasaki, 851-2213 Japan; 3 Veterinary Research Center, Nihon University, Kanagawa, 252-0880 Japan

**Keywords:** Agricultural genetics, Genotype

## Abstract

Studies using genome-wide single nucleotide polymorphisms (SNPs) have become commonplace in genetics and genomics, due to advances in high-throughput sequencing technologies. Since the numbers of required SNPs and samples vary depending on each research goal, genotyping technologies with high flexibility in the number of SNPs/samples and high repeatability have been intensively investigated. For example, the ultrahigh-multiplexed amplicon sequencing, Ion AmpliSeq, has been used as a high-throughput genotyping method mainly for diagnostic purposes. Here, we designed a custom panel targeting 3,187 genome-wide SNPs of fugu, *Takifugu rubripes*, and applied it for genotyping farmed fugu to test its feasibility in aquaculture studies. We sequenced two libraries consisting of different pools of individuals (*n* = 326 each) on the Illumina MiSeq sequencer. Consequently, over 99% target regions (3,178 SNPs) were amplified and 2,655 SNPs were available after filtering steps. Strong correlation was observed in the mean depth of coverage of each SNP between duplicate runs (*r* = 0.993). Genetic analysis using these genotype data successfully detected the known population structure and the sex determining locus of fugu. These results show the method is superior in repeatability and flexibility, and suits genetic studies including molecular breeding, such as marker assisted and genomic selection.

## Introduction

Single-nucleotide polymorphisms (SNPs) are valuable genetic markers due to their abundance and relatively uniform distribution in the entire genome. Thus, they play important roles in wide-range of research fields from basic to applied science. Recent advances in next generation sequencing (NGS) technologies enable us to genotype SNPs across the genome. However, genotyping whole-genome SNPs still remains cost-prohibitive and the number of SNPs genotyped is often excessive for several genetic analyses^1^. For instance, a relatively small to medium number of SNPs information (from hundreds to thousands) is enough to answer ecological and conservation-related questions e.g., population structure, inbreeding and genomic diversity^1,2^. In such studies, genotyping a large number of samples is more important to maximize statistical power rather than only increasing SNP information. Therefore, researchers have often sought genotyping methods which maximize the balance between cost, sample size and the number of SNPs.

Reduced representation methods, where only a fraction of the whole genome is sequenced on NGS and genotyped, will be an effective technique to increase this balance^1–5^. Here, a small subset of the genome (1% or less) is sequenced, thus sample number can be increased per run. Most familiar reduced representation methods would be genotyping-by-sequencing technologies, including restriction site-associated DNA sequencing (RADseq)^1,2,6^. In these methods, regions adjacent to restriction enzyme (RE) cut sites across the genome are sequenced and genotyped without need of any prior genomic information. The number of SNPs can be partly customized using different combinations of RE^7^. This allows reduction in genotyping cost per sample and increases the number of samples per run. RADseq have an innate weakness, that the robustness of data is often diminished when RE sites include *de novo* mutations, and/or fragmented DNA is used (*i.e*., allele dropout)^8–10^. This uncertainty becomes prominent with increased sample size, due to increases in probability of mutations in RE sites and/or in the number of samples with low DNA quality. Although such disadvantages are realized, RADseq has rapidly become popular in the field of ecological and conservation genetics, and recently also agricultural genomics^1,2,11–13^. Meanwhile, polymerase chain reaction (PCR)-based reduced representation methods, e.g. Genotyping-in-Thousands by sequencing (GT-seq)^14^, Multiplexed PCR Targeted Amplicon sequencing (MTA-seq)^15^, and Highly Multiplexed Amplicon sequencing (HiMAP)^16^, have also developed further. These approaches take advantages of PCR; only target SNPs can be consistently amplified using specific PCR primers resulting in not only high repeatability of data but more robustness against *de novo* SNPs. These PCR-based methods allow using low quality DNA^15,16^. Additionally, flexibility in the number of SNPs genotyped can be accomplished by controlling the number of primers. Today, such PCR-based reduced representation approaches are easily accomplished using commercially provided kits, namely Ion AmpliSeq technology (Thermo Fisher Scientific Inc). Presently, the AmpliSeq technology gains an advantage over other PCR-based genotyping methods in both multiplicity in target regions (up to 6,144 loci can be amplified simultaneously in a single tube), and flexibility in the balance between the number of SNPs and samples per analysis. The flexibility would be better suited over a wide range of genetic studies, including agriculture, although the technology is currently mainly used for diagnostic purposes allowing detection of genetic variances causing disorders.

In this study, we assessed the availability of the amplicon sequencing technology for genetics and genomics in aquaculture using cultured populations of the tiger pufferfish, or fugu, *Takifugu rubripes*. Fugu is one of the more valuable commercial fish in Japan^17^. Broodstock management and seed production have already been established^18–20^, and aquaculture production constitutes approximately 90% of overall production of this species^21^. In addition, fugu has been used as a model organism for comparative genomics due to its small genome size^22–24^, and extensive genomic resources including an integrative physical map are available^25–30^. This makes it easy to construct a SNP marker panel optimized for the amplicon sequencing technology. Genotyping SNPs across the genome is the primary step for molecular selective breeding, such as marker assisted selection (MAS) and genomic selection. Easy acquisition of genome-wide SNP information without large investment, such as in development of a SNP chip, will facilitate molecular selective breeding in aquaculture^6,31,32^.

To show the availability of the amplicon genotyping system, we firstly identified genome-wide SNPs in wild fugu samples by whole genome resequencing and listing of approximately 3,000 target SNPs. Secondly, we designed highly multiplexed primers using the Ion AmpliSeq Designer. After amplifying the target regions with 652 cultured individuals, we constructed two multiplexed libraries including 326 individuals each for the Illumina MiSeq sequencer. The two library pools were sequenced separately to examine the repeatability of sequencing data. Finally, we performed a population structure analysis and genome-wide association study to identify the sex determining gene, *Amhr*2^26^. Our results demonstrated the feasibility of the method in aquaculture, for both genetic and genomic study.

## Results

### SNP identification and selection for custom AmpliSeq panel

We determined the size of the AmpliSeq panel based on the throughput of the Illumina MiSeq sequencer. Using the Illumina MiSeq Reagent v2 kit (300 cycles), the sequencer produces over 4.5G base per run under paired-end mode. Since the average length of the target amplicon is 150 bp, the throughput is roughly equivalent to one million amplicons with an average of 15× depth of coverage (one million amplicons × 300 base × 15 depth = 4.5 Gb). Thus we designed approximately 3,000 SNPs, with which 326 samples (3,000 × 326 = 978,000 SNPs) can be sequenced in a single run. With our protocol, genotyping cost, including synthesis of primer pool, library preparation, and sequencing is approximately 40 USD per sample (0.013 USD per SNP).

**Figure 1:**
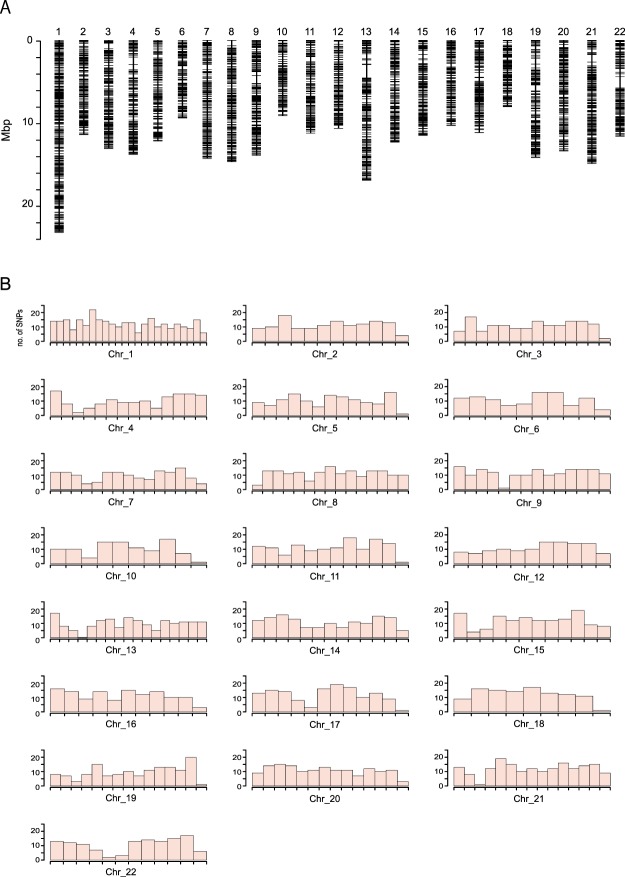
Distribution of the targeted 3,187 SNPs designed for the multiplexed PCR. (**A**) Illustration of the SNP location on each of 22 chromosomes with a scale (the vertical line on the left side of the figure). (**B**) The distribution of SNPs across the chromosomes. The x-axis shows one million base pairs interval and the y-axis shows the number of SNPs residing in each interval.

In order to construct the custom AmpliSeq primer panel, we first created a list of genome-wide SNPs collected from 20 wild-caught fugu. The summary statistics for Illumina sequencing and SNP detection are listed in Supplementary Table S1. A total of 3,232,903 putative SNPs were identified from an average of 42.6 million quality filtered reads per sample. Subsequently, we applied multiple filtering steps (described below) to select approximately 3,000 SNPs covering the whole genome. Firstly, SNPs for which genomic positions are unknown and those near the known repeat elements on the reference sequence of fugu were eliminated (UCSC table browser assembly Oct. 2011 (FUGU5/fr3)^25^). The resulting SNPs with minor allele frequency less than 0.2 and average depth over 60× were excluded. After these filtering steps, 528,285 SNPs survived. To further narrow the number of SNPs, 5,065 SNPs were randomly selected using *SelectVariants* option of GATK. In addition, SNPs near homonucleotides and/or microsatellite regions were manually excluded and then selected with a distance of roughly 20,000 bp between the neighboring SNPs, resulting in 3,329 SNPs. Finally, a custom AmpliSeq panel targeting 3,187 loci was designed by Ion AmpliSeq Designer. The resulting 3,187 SNPs were almost uniformly distributed over each of 22 chromosomes (Fig. 1A,B). Physical distances between neighboring SNP markers were 13,014–1,618,401 bp (median: 69,472 bp).

### Repeatability of sequence data from the custom AmpliSeq panel

**Figure 2:**
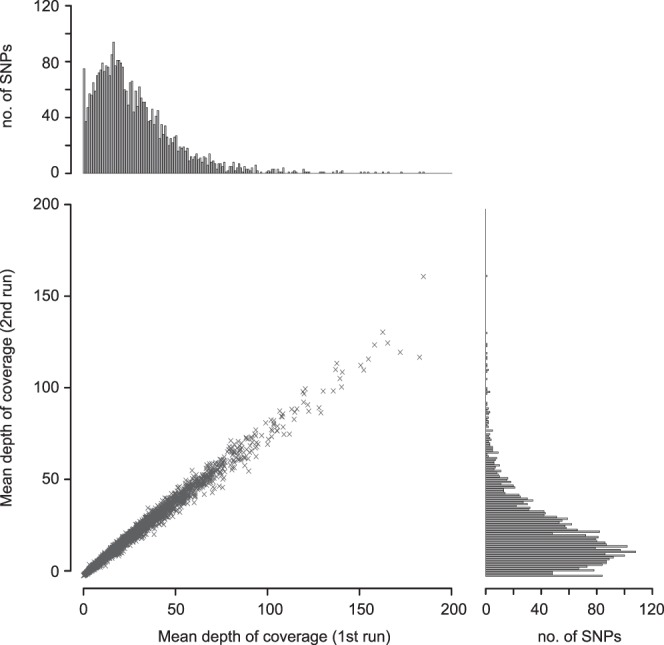
Repeatability of genotyping data from the custom AmpliSeq panel between two independent sequencing. Plots show the mean depth of each targeted SNP, indicating high repeatability of genotype data, *r* = 0.993. Histograms show distribution of depth of each targeted SNP from two runs. The mean depth of all targeted SNPs was 28.2× and 23.2× in the first and second runs, respectively.

To examine the repeatability of sequence data from the custom AmpliSeq panel, two multiplexed libraries including each of 326 cultured individuals were constructed and sequenced independently on the Illumina MiSeq Sequencer (300 bp, paired-end). As a result, 30.7 million (mean: 94,171 per sample) and 25.8 million (mean: 79,144 per sample) reads were obtained from the first and second run, respectively. After quality trimming and mapping, an average of 89,987 (95.7%) and 73,999 (94.1%) reads per sample were mapped on the genomic regions. In each run, 495 and 402 reads on average were multi-mapped on the other regions, *i.e*. secondary alignment. For the following analysis, we used only primary alignment reads (Supplementary Table S2). In total, 3,178 regions (99.7%) out of the targeted 3,187 regions obtained at least one amplicon, but the remaining nine regions had no amplicon in both sequencing runs. The mean depth of target SNPs was 28.2× and 23.2×, in the first and second runs, respectively (Fig. 2). The mean depth at each locus showed high correlation between the two sequencing runs (*r* = 0.993, Fig. 2, Supplementary Tables S3 and S4). The number of detected target loci in the first and the second run were 3,112 loci (97.6%) and 3,103 loci (97.3%) at minimum mean depth of 1×, and 2,850 loci (88.0%) and 2,789 loci (87.5%) at minimum mean depth of 6×, respectively (Supplementary Tables S3 and S4). The numbers of available loci at different levels of percentage of missing data are shown in Supplementary Fig. S1.

### Population structure and genome-wide association assay

Out of 652 individuals, 456 individuals were visually sexed and used for the following analysis to assess the availability of the genotyping method for genetic studies. To do so, we applied further filtering steps to retain only biallelic SNPs with less than 30% missing data and mean depth ≥6, and thus finally obtained 2,655 available SNPs (83.3%). Out of the lost 532 loci, 29 (1.0%) were monoallelic, 79 (2.4%) were multiallelic (≥3 alleles), 24 (0.8%) were insertion/deletion, and the remaining 400 SNPs did not fulfill the other criteria; i.e. they have >30% missing data and/or mean depth <6. These surviving 2,655 SNPs were used for the following analysis.

**Figure 3:**
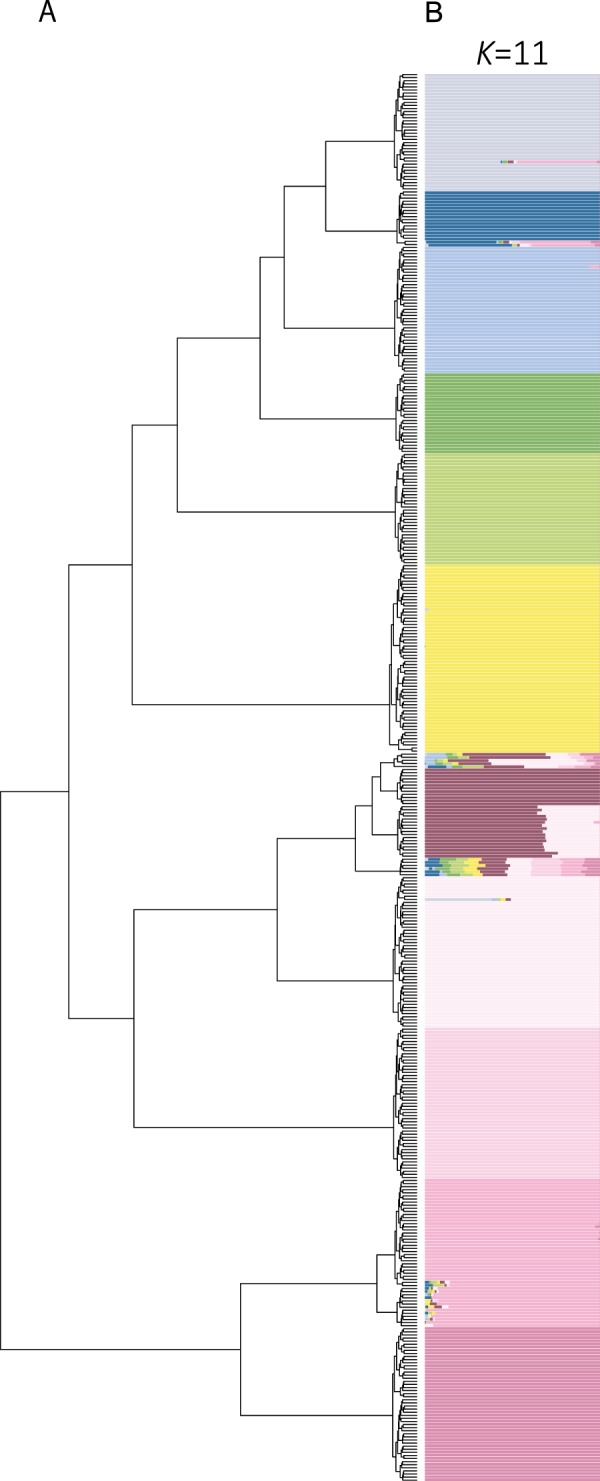
Genetic population structure of cultured population. (**A**) Ward’s hierarchical clustering based on Euclidean distance between genotypes. (**B**) The admixture proportions of individuals by estimating ADMIXTURE analysis. Each color represents the inferred genetic contribution from ancestral population (*K* = 11).

To uncover the genetic structure of the cultured population, we did hierarchical clustering and ADMITXURE analysis (Fig. 3). The optimal number of *K* was estimated as 11 based on cross validation (Supplementary Fig. S2), and the resulting subpopulations agreed with the pattern of clustering. The number of subpopulations almost corresponded with the fact that the cultured populations consist of at least 10 full-sib families (Supplementary Fig. S3).

**Figure 4:**
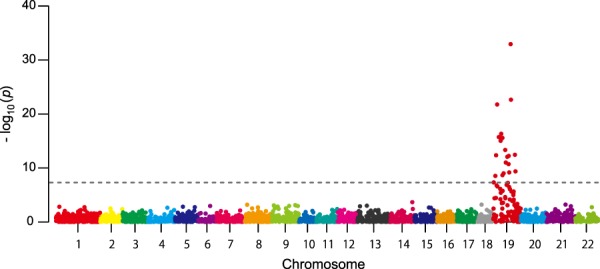
Genome-wide association assay for phenotypic sex. Manhattan plot of SNPs associated with the phenotypic sex. All SNPs above the dashed line are significantly associated with the sex. The dashed line represents genome-wide significance, *p* < 5.2 × 10^−8^. The SNP with the highest *p*-value, *p* = 1.2 × 10^−33^, located adjacent to the sex determination SNP in *Amhr2* of fugu^26^.

To further evaluate the applicability of this method, a genome-wide association study was done to find SNPs associated with phenotypic sex of fugu whose sex determination SNP had been identified (SNP_7271 on *Amhr2*)^26^. Comparison of allele frequencies between female and male with Fisher’s exact test detected SNPs associated with sex on Chr_19, corresponding to the sex chromosome of this species. Notably, a SNP closest to the sex determining SNP (136,000 bp apart from the causal SNP in *Amhr2*) showed the highest association (*p*-value = 1.2 × 10^−33^) (Fig. 4).

## Discussion

In this study, we used the PCR-based NGS method, namely the AmpliSeq technology, to construct a reduced representation library for fugu. We designed a highly multiplexed primer pool capable of amplifying approximately 3,000 SNPs across the genome in a single PCR tube. The resulting genotype data were used to validate the applicability of this method in aquaculture genetics and genomics. Here, we demonstrated that the amplicon sequencing technology could simultaneously amplify >99% of the target SNPs and produce highly repeatable genotyping data in 652 individuals. Furthermore, a standard population genetic analysis identified the known population structure and a genome-wide association study (GWAS) successfully detected SNPs associated with the sex. Notably, a SNP showing the highest *p*-value was located near the sex determination gene, *Amhr2*^26^. These results demonstrate the feasibility in aquaculture of this method for not only a standard population genetic study but also genomic studies.

In this study, we obtained approximately 82,000 raw reads per sample with 326 individuals from a single Illumina MiSeq sequencing run. On average >95% of the raw reads were mapped onto the target regions and used for the following SNP calling. Out of 3,187 targeted SNPs, 3,178 SNPs (99.7%) obtained at least one amplicon and 2,820 SNPs (88.5%) had ≥6× mean depth in 652 individuals. To validate the repeatability of sequence data, we compared the mean depth at each SNP site between two independent sequencing runs. As a result, the depth of coverage was highly correlated between two independent runs (*r* = 0.993). These results clearly demonstrate that the amplicon sequencing technology can repeatedly collect SNP data from a consistent fraction of the genome with little loss of data even while increasing the number of individuals sequenced. Taking into account the low genotyping cost (40 USD per sample or 0.013 USD per SNP), this allows us to genotype efficiently and increase sample size within a limited budget, contributing to improvement in the overall cost-effectiveness and productivity in various research scenarios. Contrary to RE based reduced-representation approaches^1,2,6,7^, such as RADseq, one limitation of this method is requirement of a reference genome and a high-density SNP list to design PCR targets. However, sequencing costs have been decreasing and various new bioinformatics tools have been developed. Therefore, acquisition of whole genome sequences is facilitated^33,34^ and this limitation will not be a critical problem in the near future.

After several filtration steps, 2,655 out of 3,187 SNPs were retained from 456 phenotyped individuals. Among the filtered-out 532 SNPs, 400 were removed due to insufficient depth of coverage. These SNPs could be retrieved by reducing the number of samples applied to the MiSeq sequencer (24–30 million reads per run) or using other NGS sequencing platforms with larger output, such as Illumina HiSeq X (three billion reads per lane). On the other hand, 132 SNPs were eliminated because these were either monoallelic, multiallelic (≥3), or insertion/deletions. The absence of these SNPs would be caused by differences in genetic background between the wild and cultured populations, because we designed the SNP panel based on the genome-wide resequencing using wild populations. Designing the SNP panel based on the target population will help prevent this loss of SNP data.

Genomic data is increasingly important in aquaculture selective breeding programs^12,35–37^. Molecular-based selective breeding will be broadly separated into two categories: marker-assisted selection (MAS) and genetic selection (GS). MAS incorporates information on only polymorphisms significantly associated with the target trait via QTL analysis or GWAS. In general, the density of the designed SNP panel (3,187 SNPs) is relatively small for GWAS, which typically often requires a large number of SNPs, from thousands to several hundred thousand^1^. Meanwhile, the marker density for GWAS depends on the extent of linkage disequilibrium (LD) between SNP markers and causative polymorphisms in loci affecting target traits (QTL)^38^. Since breeding populations are usually recently admixed, large LDs are expected to be introduced in the aquaculture populations^39,40^. Therefore, hundreds of thousands of markers may not always be required in such populations, because most of LD could be captured by a relatively low or moderate density of SNP markers^40^. In this study, the marker density was decided based on the size of the genetic map and the marker intervals were designed with almost equal spacing (Fig. 1) which could benefit detection of QTL in GWAS. In the case of fugu, the genetic map of male and female spans 1,202.8 and 2,189.8 cM, respectively^25^, and thus the 2,655 SNPs, the number of SNPs finally obtained after quality the filtering steps, distributed on average 1.6 SNPs/cM. As expected, we could detect a SNP strongly associated with the sex and located adjacent to the sex determination gene, *Amhr2*^26^. This result indicated that the marker density and distribution might be sufficient to detect QTLs with large effects in the cultured populations. However, further studies are needed to confirm if the SNP density is high enough to detect small/medium effect QTLs from a population with a more complicated structure. The number of multiplexed primers could be easily increased if necessary because the AmpliSeq technology presently offers dozens to six thousand targets in a single primer pool. Target SNPs can be further increased by mixing PCR products amplified using other primer pools. This flexibility allows us to efficiently make adjustment of SNPs numbers for analysis.

GS can incorporate all SNP markers regardless of statistical significance^41,42^. Therefore, when polygenic traits underpinned by many genes with minimal effects are targeted, higher genetic gain will be accomplished with GS compared to MAS^41^. The accuracy of GS depends on the extent of LD as well as the power of GWAS^38^. In this study, we successfully detected SNPs associated with sex phenotype, suggesting that most of LD was covered by the moderate density SNP panel, and thus the panel may also be available for GS. In farmed Atlantic salmon, high prediction accuracy for polygenic traits, e.g., sea lice resistance and growth, have been achieved with moderate density of SNPs (5,000 SNPs, equivalent to 2–3 SNPs/cM)^35,36,40,43^, a similar density to our study (1.6 SNPs/cM). These results suggest that GS can be done using genotyping by targeted amplicon sequencing with several thousand SNPs in the cultured fugu populations. Taken together, our results suggest that the amplicon sequencing technology based on AmpliSeq custom panel offers high cost-efficiency, flexibility in sample/SNP size and repeatability in the number of genotyped SNPs. Thus, the approach will be available for molecular breeding in aquaculture species, leading to increased exploitation of genetically improved fish carrying desirable traits.

## Materials and Methods

### Whole-genome resequencing

We resequenced twenty commercially wild-caught fugu for genome-wide SNP scanning. Genomic DNA was extracted from the caudal fin using a Gentra Puregene tissue kit (Qiagen, Hilden, Germany) according to the manufacturer’s instruction. Library construction and genome sequencing was done by Hokkaido System Science Co. (Hokkaido, Japan). The library was constructed for each sample using TruSeq Nano DNA LT Sample Prep Kit (Illumina, CA, USA) following the manufacturer’s regular protocol (insert size 350 bp). Ten samples were loaded per lane and sequenced 100 bp from both ends on the Illumina Hiseq 2000 sequencer (Illumina).

Raw reads were quality-trimmed using Trimmomatic^44^ v0.35 by setting the parameters as follows; ILLUMINACLIP TruSeq3-PE-2.fa:2:30:10, LEADING:19, TRAILING:19, SLIDINGWINDOW:30:20, AVGQUAL:20, and MINLEN:101. After trimming, reads survived in both ends were mapped onto the reference sequence of fugu^25^ (GenBank: CAAB00000000.2) using BWA-mem^45^ with default parameter. The resulting alignments were passed to the local realignment steps with *RealignerTargetCreator* and *IndelRealigner* commands of GATK^46,47^ version 3.3 and PCR duplicates were marked with Picard-1.114 (available at http://broadinstitute.github.io/picard). Initial variant site calling was performed per sample using GATK-UnifiedGenotyper. Variant sites shared by all individuals were extracted by the *merge* option of BCFtools^48,49^ v1.2 (available at https://github.com/samtools/BCFtools) and used as the confident sites for base quality score recalibration step of GATK v3.3. The base quality score recalibration was done for four cycles, and base-recalibrated BAM files were created. The second variant site calling was done per sample on the base-recalibrated BAM file using GATK-Haplotypecaller with–standard_call_conf 30 and–standard_emit_conf 30 options to generate gVCF files. Variant sites were genotyped using GATK-GenotypeGVCFs to create the final SNP list.

### SNP selection for custom AmpliSeq panel

In this study, 3,000 SNPs were selected as targets for custom AmpliSeq panel (Life Technologies, CA, USA) from the final SNP list according to the following procedures. At first, SNPs locating in 200 bp upstream and downstream of the known repeat on the reference sequence of fugu (UCSC table browser assembly Oct. 2011 (FUGU5/fr3)^25^) and insertion/deletion in the SNP list were removed. Of the remaining SNPs, those with the minor allele frequency less than 0.2 and average depth of ≥60× were removed using VCFtools^50^ version 4.1. In addition, the SNPs mapped on the scaffolds with unknown genomic position or orientation were excluded. To narrow down the number of targeting SNPs, 1.25% of the SNPs were randomly selected using *SelectVariants* of GATK-v3.6. In addition, the regions covering 200 bp upstream and downstream of SNPs that included homonucleotide sequences (≥10 bp of poly-A, poly-T, poly-G and poly-C) and/or microsatellite (CA/GT repeats) were manually removed. Then, the neighboring SNPs were separated by roughly 20,000 bp. Finally, the position information of the resulting SNPs was used to create a custom AmpliSeq panel designed by the Ion AmpliSeq Designer system; the range of amplicon size is 125–175 bp (ThermoFisher: https://www.ampliseq.com accessed Sep. 2016). As a result, we obtained 3,187 primer pairs.

### Library construction and amplicon sequencing

Applicability of the designed custom AmpliSeq panel was validated using 652 individuals including at least 10 full-sib families purchased from six aquaculture farms in Nagasaki prefecture. Two independent libraries with 326 individuals each were constructed and sequenced separately. Among 652 individuals, 456 individuals were dissected for visual sex determination for the following genome-wide association assay (GWAS). Genomic DNA extraction was done as mentioned above. DNA concentration was measured using a UV spectrophotometer (BioPhotometer; Eppendorf, Hamburg, Germany).

The first PCR was performed on genomic DNA with the custom AmpliSeq primer pools. The PCR reaction mixture contained 40 ng of genomic DNA, 9 µl of 2× AmpliSeq primer pool, 10 µl of 2× Multiplex PCR Buffer (Multiplex PCR Assay Kit ver.2, Takara Bio Inc., Shiga, Japan), and 0.2 µl of Multiplex PCR Enzyme Mix, in a final volume of 20.2 µl. The first PCR condition was as follows: initial denaturation at 94 °C for 1 min; 20 thermal cycles of denaturation at 94 °C for 30 s, primer annealing and extension at 60 °C for 4 min; followed by a final incubation at 72 °C for 10 min. PCR amplicons were digested with USER enzyme (New England Biolabs Inc., MA, USA) at 37 °C for an hour and purified with 1.8× volume of Agencourt AMpure XP (Beckman Coulter, Inc., CA, USA). The PCR products were end repaired, dA-tailed, and ligated with the NEBNext Adaptor for Illumina using NEBNext Ultra Library prep reagents (New England Biolabs Inc.) according to the manufacturer’s instruction. The adaptor-ligated products were purified twice with AMpure XP purification (1.4× vol. each). Then, P7/P5 adaptors including custom-designed 8 bp dual indices (adopted from Meyer and Kircher)^51^ were added as primers by a second PCR. The second PCR reaction mixture contained 6 µl of adaptor-ligated DNA products, 2 µl of each 10 µM index primer, and 10 µl of NEBNext Q5 Hot Start HiFi PCR Master Mix (New England Biolabs Inc.), in a final volume of 20 µl. The second PCR condition was as follows: initial denaturation at 98 °C for 30 sec; 7 thermal cycles of denaturation at 98 °C for 10 s, primer annealing and extension at 65 °C for 75 sec; followed by a final incubation at 65 °C for 6 min. The resulting PCR products were purified with AMpure XP (1.1× vol.). The total 326 barcoded libraries were mixed with equal concentration. The final concentration of the multiplexed library was quantified by quantitative PCR using NEBNext Ultra Quant Kit (New England Biolabs Inc.) and diluted to 8.5 pM mixed with ~14% PhiX (PhiX Control V3; Illumina). The two multiplexed libraries, consisting of 326 samples each, were sequenced separately on the Illumina MiSeq with MiSeq Reagent Kit v2 (300 cycles, Illumina).

### Genotype calling

Raw reads that passed an Illumina purity filter were demultiplexed based on the index sequences and split into each FASTQ file using Generate FASTQ workflow on the MiSeq platform. The raw reads were quality-trimmed using Trimmomatic by setting the parameters as follows: ILLUMINACLIP TruSeq3-PE-2.fa:2:30:10, LEADING:19, TRAILING:19, CROP:146, HEADCROP:5, SLIDINGWINDOW:30:20, AVGQUAL:20, and MINLEN:60. After trimming, the reads surviving both of the pair were mapped onto the subset of the reference sequences covering 50 bp upstream and downstream of the targeted loci. Reads were marked as “secondary alignment”, mapped on multiple regions, were excluded and only primary alignments were retained using SAMtools flag command (SAM flag = 256). Subsequently, genotype calling was done by jointly analyzing 652 individuals using GATK v4.0. Briefly, gVCF files were generated for each sample with GATK-HaplotypeCaller and merged into a single gVCF file with GATK-CombineGVCFs command. Joint genotyping was performed with GATK-GenotypeGVCFs^52^.

### Repeatability of results from custom AmpliSeq panel

Mean depth of coverage at each targeted SNP was analyzed using BEDtools^53^ *coverage* command and Pearson’s correlation coefficient was calculated between the two sequencing runs.

### Population structure and genome-wide association assay

To assess the applicability of this custom AmpliSeq panel for genetic studies, we applied this system to analyses of standard population genetics and GWAS for sex using the 456 cultured individuals with records of phenotypic sex. The genotyping data were further filtered based on the following criteria using VCFtools v4.1, (1) at least 70% of individuals were called, (2) only two alleles were present, and (3) average depth was ≥6. Subsequently, we applied LinkImpute^54^ v1.1.3 to infer missing genotype data and the resulting SNPs with minor allele frequency (<5%) were further filtered out using VCFtools.

The population structure of the sample populations was inferred by means of hierarchical clustering based on Ward’s method with Euclidean distances using *hclust* on R^55^. Maximum likelihood estimation of individual ancestries was done using ADMIXTURE version 1.3.0^56^. To choose the optimal number of ancestral populations (*K*), we performed five-fold cross validation for *K* from 1 to 20 following the manual. GWAS for the phenotypic sex (male phenotype as case and female as control) was performed with dominant model using PLINK v1.07^57^. A Manhattan plot was drawn using the “qqman” software package^58^ on R.

### Ethics

All experiments described herein were approved by and carried out in accordance with the IACUC (Institutional Animal Care and Use Committee) of the Graduate School of Agricultural and Life Sciences, University of Tokyo (P-170529004). All methods were performed in accordance with the IACUC guidelines and regulations.

## Supplementary information

Supplementary Figures

Supplementary Tables

## Data Availability

The resequencing data and the amplicon sequence reads have been deposited in the DDBJ Sequence Read Archive (Submission: DRA007457-DRA007464; BioProject: PRJDB7538; BioSample: SAMD00143641-SAMD00144312).
