# Supplementary material for: A highly flexible and repeatable genotyping method for aquaculture studies based on target amplicon sequencing using next-generation sequencing technology
Source: Sci Rep. 2019 May 6;9:6904. doi: 10.1038/s41598-019-43336-x (PMC6502806; doi:10.1038/s41598-019-43336-x)
Supplement: Supplementary file 1 — Supplementary Figures [file 41598_2019_43336_MOESM1_ESM.pdf]

Supplementary information

A highly flexible and repeatable genotyping method for aquaculture studies based on target amplicon sequencing using next-generation sequencing technology

Mana Sato[1], Sho Hosoya[1]*, Sota Yoshikawa[1,2], Shun Ohki[1], Yuki Kobayashi[3], Takuya Itou[3], Kiyoshi Kikuchi[1]

[1]Fisheries Laboratory, University of Tokyo, Hamamatsu, 431-0214, Japan.
[2]Seed production Technology Development Center, Nagasaki Prefectural Institute of Fisheries, Nagasaki, 851-2213, Japan
[3]Veterinary Research Center, Nihon University, Kanagawa, 252-0880, Japan

*Correspondence.
Sho Hosoya
E-mail address: ahosoya@mail.ecc.u-tokyo.ac.jp

Supplementary Figures S1-S3

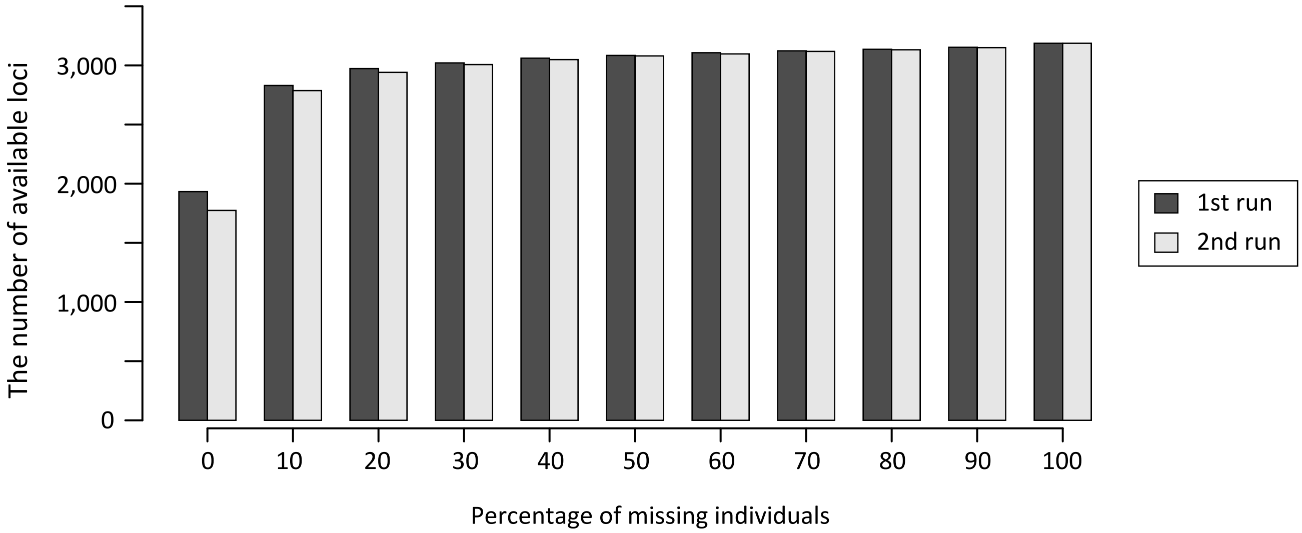

Supplementary Figure S1. The number of available loci with different levels of missing rates of genotyped individuals. Zero-percent indicates the number of available loci with no missing data, whreas the number of available loci with 100% missing is equivalent to the total number of targeted loci, i.e. 3,187 loci.

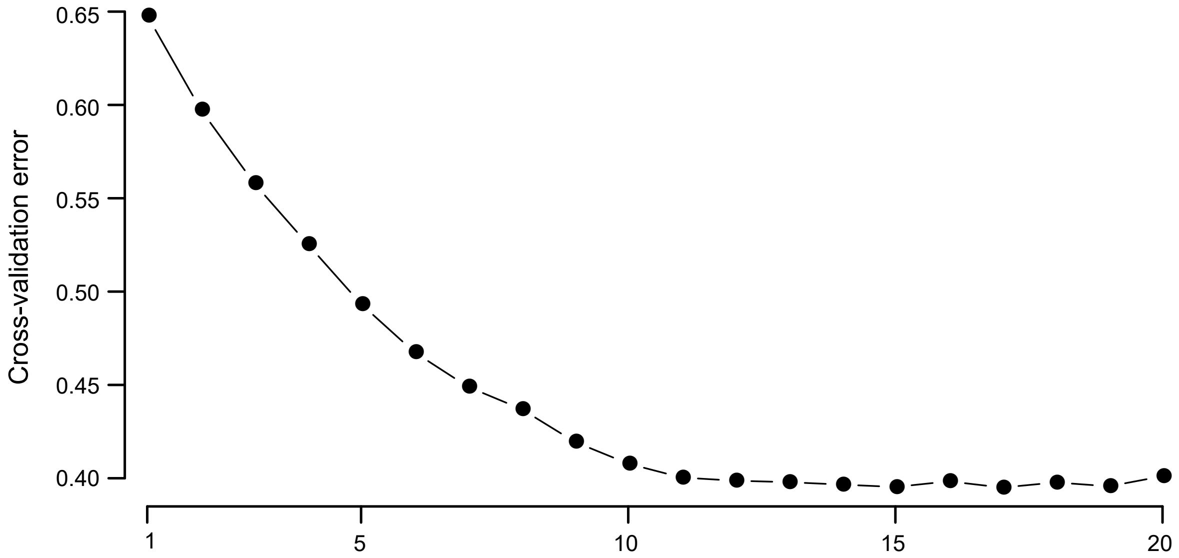

Supplementary Figure S2. Plot shows the error rate estimated by cross validation test for each *K* in the ADMIXTURE analysis from *K*=1 through *K*=20.

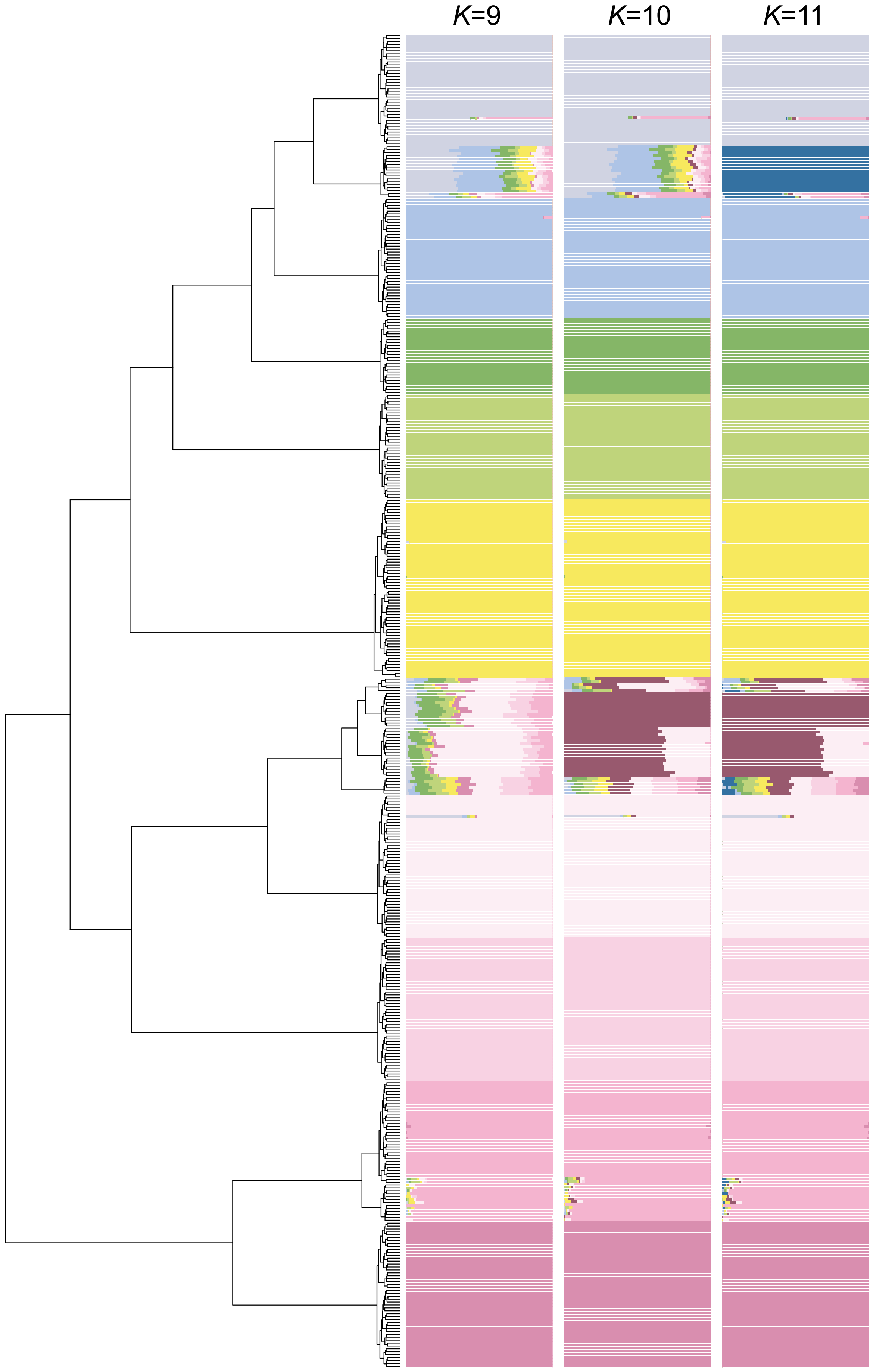

Supplementary Figure S3. Hierarchical clustering and population structure plots of the cultured population. The admixture proportions of individuals were estimated by ADMIXTURE software (*K*=9 to *K*=11)
